# The genetic editing of *GS3* via CRISPR/Cas9 accelerates the breeding of three-line hybrid rice with superior yield and grain quality

**DOI:** 10.1007/s11032-022-01290-z

**Published:** 2022-04-08

**Authors:** Juan Huang, Lijun Gao, Shuming Luo, Kaiqiang Liu, Dongjin Qing, Yinghua Pan, Gaoxing Dai, Guofu Deng, Changlan Zhu

**Affiliations:** 1 Key Laboratory of Crop Physiology, Ecology and Genetic Breeding, Ministry of Education, Jiangxi Agricultural University, Nanchang, 330045 China; 2 Guangxi Crop Genetic Improvement and Biotechnology Laboratory/Guangxi Key Laboratory of Genetic Improvement of Crops, Nanning, 530007 China; 3 Flower Research Institute, Guangxi Academy of Agricultural Sciences, Nanning, 530007 China; 4 Guangxi Academy of Agricultural Sciences, Nanning, 530007 China; 5 Rice Research Institute, Guangxi Academy of Agricultural Sciences, Nanning, 530007 China

**Keywords:** Grain size, CRISPR/cas9, Yield, Grain appearance, Three-line hybrid rice

## Abstract

Grain size is one of the major traits that determine rice grain yield and quality. The *GS3* gene is the first major quantitative trait locus (QTL) that was identified in regulating rice grain length and weight. It was reported that the *gs3* allele with a mutation in the organ size regulation (OSR) domain of the *GS3* protein produced longer grains. In this study, we used the CRISPR/Cas9 gene editing technology to introduce an edited *gs3* allele into our *indica* maintainer line, Mei1B, to enhance its grain yield and quality. Through molecular analysis and sequencing, a homologous edited-*gs3* mutant line without any transgene was obtained in the T_1_ generation and was named Mei2B. A superior male sterile line Mei2A was generated by backcrossing the cytoplasmic male sterile (CMS) line Mei1A with Mei2B. Mei2B had a higher grain quality and yield compared to its wild-type Mei1B. Its grain length increased by 7.9%, its length/width ratio increased from 3.89 to 4.19, TGW increased by 6.7%, and grain yield per plant increased by 14.9%. In addition, genetic improvement of other quality traits including brown rice length (6.83 mm), brown rice grain length/width ratio (3.61), matched the appearance standards set for traditional Simiao (silk seedling) type cultivars. Two restorer lines were outcrossed to both Mei1A and Mei2A to produce hybrid rice. Compared to two hybrids of Mei1A, the hybrids of Mei2A had longer grains, higher length/width ratio, TGW, and yield per plant. In addition, the hybrids of Mei2A showed a better grain appearance including better translucency, a lower chalky rice rate, and degree of chalkiness than the hybrids of Mei1A. These results demonstrated that the introduction of an elite *gs3* allele into Mei1A via CRISPR/Cas9 gene editing technology led to significant genetic improvement of the rice grain. The resultant CMS line Mei2A(*gs3*) displayed much higher grain quality and yield than the original Mei1A. Therefore, our study demonstrated that the targeted genetic improvement via gene editing technology can enhance rice breeding, especially the breeding of three-line hybrid rice.

**Supplementary Information:**

The online version contains supplementary material available at 10.1007/s11032-022-01290-z.

## Introduction

Rice is a staple cereal crop grown in more than 100 countries across the world and supplies calories for 2/3 of the world’s population. The traits related to grain size (GS) including grain length (GL), grain width (GW), grain thickness (GT), and grain length/width ratio are important factors determining rice yield(Sakamoto and Matsuoka 2008). These traits are also closely related to rice quality characteristics such as grain appearance, processing, cooking, and taste (Xu et al. 1993, 2004; Tan et al. 2000; Li et al. 2004; Song et al. 2007; Shomura et al. 2008; Wang et al. 2008; Wang et al. 2015; Huang et al. 2013; Huang and Qian 2017). During grain development, especially the filling process in big and round grain, it is easy to generate chalkiness because of the long transportation route from the back to the abdomen in the grain itself. Therefore, the transparent and high-quality rice grains are mostly produced in slender grain varieties (Gu et al. 2001; Xu et al. 2004). The market demand for high-quality slender grain rice has increased recently and has led to research focus for breeders to develop high grain length/width ratio varieties or Simiao-type varieties(grain length ≥ 6.5 mm and rice length/width ratio ≥ 3.5, T/GDSMM 001–2019) (Fitzgerald et al. 2009; Wang et al. 2012).

Traditional rice breeding methods significantly depend on the breeder’s experience since complex trait selection can be difficult as well as labor and time consuming. Although MAS (marker-assisted selection) breeding technology greatly increases breeding efficiency, it is somewhat limited when a low recombination rate exists and the genetic drags of the target gene/QTL locus are hard to break. Recently, a genome editing tool derived from an adaptive immune mechanism of microorganisms, the CRISPR-Cas9 (clustered regularly interspaced short palindromic repeats/CRISPR-associated protein9) system, has been successfully applied to plants (Li et al. 2013; Nekrasov et al. 2013; Shan et al. 2013). This technology can precisely modify a plant’s own genes without the introduction of foreign ones (Jiang et al. 2013; Liang et al. 2014; Cai et al. 2015; Lawrenson et al. 2015; Iqbal et al. 2016). Through this gene editing technology, desired traits can be quickly introduced into a target variety, thereby significantly improving the breeding efficiency (Zhang et al. 2016; Tang et al. 2017; Soyk et al. 2017; Kuang et al. 2020).

More than 500 QTL genes related to rice grain size have been identified (http://www.ricedata.cn/ Index. HTM). Among them, 19 QTL genes have been cloned in rice (Chen et al. 2020). Of these cloned QTL genes, *GS3*, *qGL3/GL3.1*, *TGW3/GL3.3*, *LGY3*, and *GS9* are major QTLs, and they have negative effects on controlling grain length (Fan et al. 2006; Qi et al. 2012; Zhang et al. 2012; Liu et al. 2018; Xia et al. 2018; Ying et al. 2018; Zhao et al. 2018). Loss-of-function at the N-terminal organ size regulating (OSR) domain of the GS3 protein made rice produce longer grains. It was found that of 78 varieties, about half of the *indica* varieties and one-tenth of the *japonica* varieties contained the long grain allele *gs3* at the GS3 locus, indicating that this gene has significant potential for rice production (Fan et al. 2006; Mao et al. 2010). Previous studies have shown that the knockout of the *GS3* gene can lead to longer grain lines. These studies mainly focused on the oval-shaped *japonica* varieties for germplasm creation, but a few focused on long-shaped *indica* rice (Han et al. 2018), especially *indica* hybrid rice (Li et al. 2016; Shen et al. 2016 and 2017; Chen et al. 2020).

The three-line hybrid rice system that includes a cytoplasmic male sterile line, a maintainer line, and a restorer line has made a significant contribution to food security in China (Yuan and Tang 1999). Until now, the breeding of three-line hybrid rice still faces great challenges because of the low efficiency in creating excellent maintainer lines and the high cost of seed production. In particular, when traditional cross-selection methods are used to introduce exogenous elite genes into improved maintainer lines and sterile lines, this is accompanied with the risk of introgressive major or minor restoring genes, which can significantly decrease the efficiency of hybrid rice breeding (Ren et al. 2016). Aimed to test whether the introduction of a *gs3* allele into an *indica* CMS (cytoplasmic male sterile) line Mei1A could improve the grain quality and yield of its hybrids, we focused on editing the *GS3* gene of this maintainer line Mei1B using CRISPR/Cas9 technology in this study. Mei1A is a derivative of an elite CMS line of MeiA, whose hybrid varieties are used to dominate the rice industry at the beginning of this century in Guangxi, China, because of the small grain size leading to better quality (Liang et al. 2001). Mei1A inherits many favorable traits from MeiA, including a high yield potential and high GCA (general combination ability). However, due to the disadvantage of its grain length, it is hard to use Mei1A as a hybrid parent to breed a competitive hybrid rice to match the current rice market demand for longer and slender (Simao-type) grains and better taste. In our research, this results showed that the introduction of a *gs3* allele into Mei1A via CRISPR/Cas9 technology did improve grain quality and yield of Mei1A’s mutant (Mei2A) and its hybrids. Such a genetic improvement system could rapidly establish superior parental lines from small grain to long grain varieties, thus laying a foundation for sustainable utilization of existing resources.

## Materials and methods

### Plant materials

Plant materials included an *indica* maintainer line Mei1B, and its corresponding sterile line Mei1A and the relationship of Mei1B and Mei1A were shown in Fig. 2a. Restorer lines Guanghui 998 (GH998) and Gui 715(G715) were used as the test-cross parents. These plant materials were grown in the transgenic isolation greenhouse of Guangxi Academy of Agricultural Sciences during the entire time of the research.

### GS3 genotype detection of Mei1B and its target sequence design

To identify whether MeilB contains an OSR mutation at the N terminus of the GS3 protein thereby introducing a target sequence mutation, two primer pairs GS3-F1/R1 and GS3-F2/R2 (Table S1) were designed near the exons 1 and 2 of *GS3* (Os03g0407400) sequence to amplify DNA fragments of the maintainer line Mei1B. The PCR products were isolated on 1% agarose gel and sequenced. The sequencing results were then compared with the *GS3* (Os03g0407400) sequence in the NCBI data base. This confirmed that the exon2 of the *GS3* gene in maintainer line Mei1B did not contain a C-A base shift in the OSR domain that caused the long grain mutation. To improve the effectiveness of detecting loss-of-function of the *GS3* gene, two gRNA target sites were further designed on exons 1 and 2 of the *GS3* gene in Mei1B via the website (http://www.rgenome.net/cas-designer/). They were target 1(cctcgaggaatccgatctcgcgg) in the exon 1 and target 2 (tgcagcatctggaggcagcgtgg) in exon 2. The sequences of these two targets were also screened for off-target effect using the BLAST program from the NCBI website. It showed that there were no matched off-target sequences, indicating an extremely low probability of any off-target effects in our experiment.

### Plasmid construction and genetic transformation

ECO31I restriction sites were introduced into the target site sequence of *GS3* with two primer pairs GS3-Y1 + /GS3-Y1- and GS3-B1 + /GS3-B1-, respectively (Table S1). The double-stranded DNA fragments were ligated into the vectors pBWA and pBWD by restriction endonuclease enzyme and ligase. The recombinant plasmid CRISPR-Cas9-GS3 is shown in Fig. S1.

To screen the positive plasmid construct, vector primers Yl-R + and Pbw2- (Table S1) were used for PCR amplification of the 1100 bp editing element in the recombinant plasmid. Amplified products were verified by sequencing and then transferred into the *Agrobacterium* EHA105. The recombinant plasmid CRISPR-Cas9-GS3 was transformed into calli of the rice variety Mei1B, according to the *Agrobacterium*-mediated transformation method. Finally, hygromycin was used to screen T_0_ transgenic lines. Positive lines were also identified by PCR amplification using a hygromycin-resistant gene primer pair (Hyg-F/Hyg-R) and GS3 primers GS3-F1/-R1, GS3-F2/-R2 (Table S1).The PCR products were sequenced. The whole transformation experiment was undertaken by the BioRun company in Wuhan, China.

### Grain size measurement of T_0_ transgenic lines

Seedlings of transgenic lines and wild-type plants were simultaneously planted at our transgenic greenhouse. Ten fully filled uniform grains of a transgenic line/wild-type plant were harvested at maturity stage and measured for grain length using an automatic grain test instrument (Wanshen SC-G, China).

### Establishment of the long-grain maintainer lines and sterile lines

To screen transgene-free mutants, Hyg-F/Hyg-R were used to amplify the DNA template of each individual line in the T_1_ generation. Additionally, an individual Mei1B line of the T_1_ generation which had the homozygous mutation of OSR significantly increased grain length, and no marker gene was named as Mei2B (Fig. 2b) and was chosen to outcross with rice sterile line Mei1A. After twice backcrossing and with selection, one sterile line with the stable long-grain trait was obtained. These new lines were identified by sequencing to confirm that they had the edited *gs3* locus and no transgene component and were named as Mei2A (Fig. 2d).

### Testing and trait characterization of potential hybrid rice combinations

The widely used restorer line GH998 together with our own long-grain restorer line G715 was used to test the improved long-grain sterile lines together with the original sterile line for hybrid rice combinations. Ten plants from each hybrid combination and their female parents and five plants from each restorer line were used to investigate the panicle length and grain number per panicle. The grain length and grain width of each line were measured on ten fully filled seeds. One hundred grain weight was randomly measured and then converted into 1000 grain weight. Rice grain quality was assessed following the standards set by the Quality for Cooking Rice Variety NY/T 593–2013. At the same time, fluorescent labeled primers were developed via the penta-primer amplification refractory mutation system (PARMS) (Ye et al. 2001; Zhang et al. 2019; Lu et al. 2020), and primers of genes related to rice grain traits such as *GS3*, *Wx*, *ALK*, and *Chalk5* were used to identify the relevant parental genotypes (Fan et al. 2006; Hirano et al. 1998; Isshiki et al. 1998; Gao et al. 2003; Li et al. 2014). The primer information is provided in Table S1. Data analyses were performed using the GraphPad Prism 8 software.

## Results

### Targeted mutation of the GS3 gene generated three edited gs3 loci

**Fig. 1:**
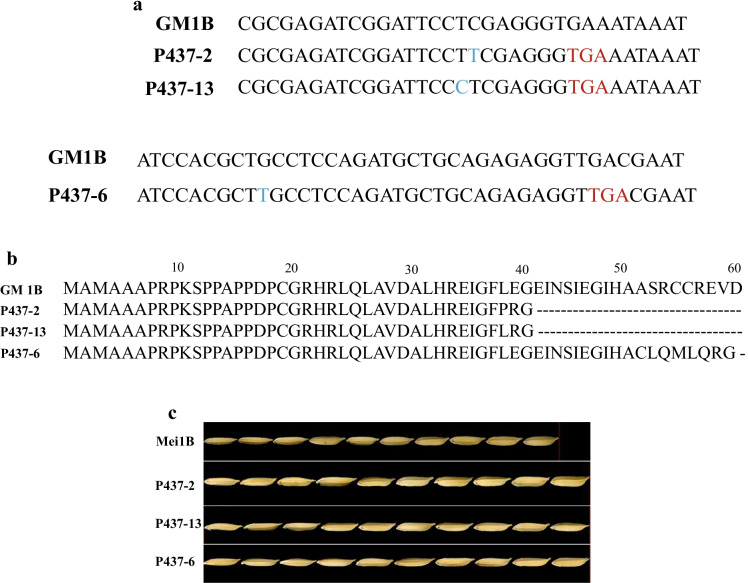
Sequencing and amino acid analyses of the three homozygous edited transgenic lines of *GS3* and their corresponding grain shape**. a** Sequencing analysis of three transgenic plants and the control of maintainer line Mei1B. Mutations with 1-bp insertions are indicated by blue letters and stop codons are marked in red. **b** *GS3* amino acid alignment of three mutants and the control Mei1B. **c G**rain length difference between the control and three mutants. Mei1B: wild-type maintainer line containing the *GS3* allele. P437-2, P437-13, P437-6: mutant maintainer lines containing the edited *gs3* allele

The CRISPR/Cas9 construct with two editing targets of the *GS3* gene were introduced into the hybrid maintainer line Mei1B, and 12 edited transgenic plants were obtained. DNA sequencing of the *GS3* gene from these 12 transgenic lines revealed that biallelic mutations occurred in three transgenic lines, the two lines (P437-2, P437-13) were homozygous at the target 1 of *gs3*, and the line P437-6 was homologous at the target 2 of *gs3* (Fig. 1). The three T_0_ transgenic plants were then self-pollinated to produce the T_1_ plants. Grain length and grain width of the three transgenic lines (T_1_) were next measured and compared with that of the control (the maintainer line, Mei1B). Phenotypic comparison showed that the average grain length of these three homozygous mutant lines (P437-2, P437-6, P437-13) were longer than 10.00 mm, while the average grain length of the control Mei1B was 9.40 mm, indicating a > 5% increase of grain length in the three mutant lines (Fig. 1, Table S2). Sequence alignments of these three homozygous mutants against that of the control MeilB further showed that 1-base pair(bp) insertion occurred at each target site, inducing gene frame shift of *GS3* and then resulting in a premature translation termination (Fig. 1a). Thus, we generated three different edited *gs3* loci which regulated the long grain trait of the rice studied.

### Identification of transgene-free maintainer line Mei2B with homozygous gs3 mutation

**Fig. 2:**
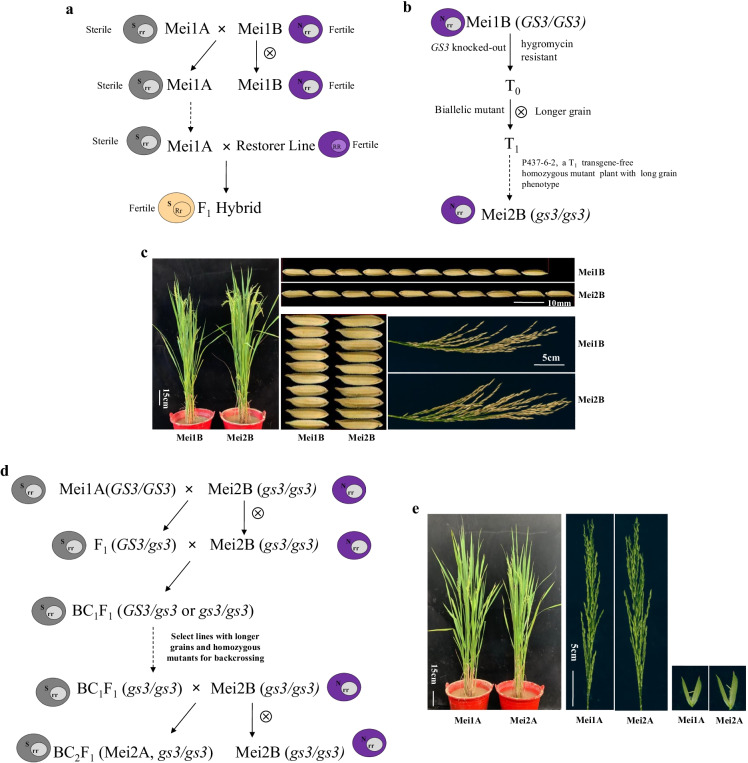
Schematic diagrams of the three-line hybrid rice system and process for convertingthe mutant maintainer line Mei2B into a mutant male sterile line Mei2A. **a** Diagram of the three-line hybrid system. It consists of a cytoplasmic sterile (CMS) line and a maintainer line and a restorer line. The CMS line carries both the cytoplasmic sterile gene (S) and the nuclear recessive sterile gene (rr) and is used to produce hybrid seed. The maintainer line carries the cytoplasmic fertile gene (N) and the nuclear recessive sterile gene (rr). It is fertile per se. When it is crossed to the CMS line, their hybrids remain sterile. So, the maintainer line is used to maintain the sterility of CMS and produce sterile seeds for hybrid seed production. The restorer line carries the dominant nuclear fertile gene (RR). When it is crossed to a CMS, its dominant nuclear gene (R) restores the fertility of their hybrids. The male sterile line Mei1A(S(rr)) and its corresponding maintainer line Mei1B (N (rr)) have the only difference in fertility. Male sterile lines rely on maintainer lines for reproduction and improvement. **b** The process to create the new maintainer line Mei2B carrying homozygous *gs3* alleles. **c** Photographs of the plant, panicle, grain length, and width of Mei1B and Mei2B. **d** The process to convertthe maintainer line Mei2B to superior long-grain sterile line Mei2A. **e** Photographs of the plant, panicle, and spikelet of Mei1A and Mei2A

The three transgenic T_0_ plants, P437-2, P437-6, and P437-13, were homozygotes at the *gs3* locus. We then selected transgene-free plants from the three transgenic plant T_1_ progenies via PCR amplification of the hygromycin-resistant gene (Fig. S2). The PCR results identified that a transgene-free plant (P437-6–2) with the nonfunctional *gs3* allele and without the selection marker gene from the T_1_ offsprings of P437-6. This transgene-free line with the nonfunctional *gs3* allele termed as Mei2B (Fig. 2b, c) would be applied to develop a new long-grain sterile line Mei2A (Fig. 2d, e).

### New sterile line Mei2A construction through the transgene-free maintainer line Mei2B

The selection process to develop the male sterile line Mei2A is presented in Fig. 2. Firstly, the maintainer Mei2B carrying the edited *gs3* loci was backcrossed to the sterile line Mei1A. Then, sterile lines with a homozygous mutation and significantly increased grain length were selected in the BC_1_F_1_ generation. The selected line was next crossed with Mei2B to produce the new sterile line Mei2A (Fig. 2d).

### Agronomic trait comparisons between Mei2B with the gs3 mutation and the control line Mei1B with the GS3 allele

**Fig. 3:**
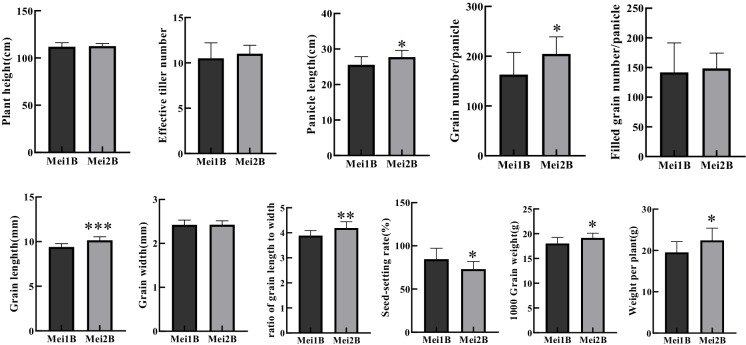
Comparison of agronomic traits between Mei1B with the *GS3* allele and Mei2B with the *gs3* allele. *, *, and *** indicated the significant difference of studied traits of Mei1B and Mei2B at *P* levels 0.05, 0.01, and 0.001. Each *p* value for each trait was obtained from a *t* test between Mei1B and Mei2B (*n* = 10 for each genotype)

To determine how the knockout of the *GS3* gene influences rice agronomic traits, the major relevant agronomic traits of maintainers Mei1B and Mei2B were investigated, including the grain length, grain width, ratio of grain length to width, panicle length, grain number per panicle, filled grain number per panicle, seed setting rate, 1000 grain weight, effective tiller number, tiller number at the active stage, plant height, and weight per plant (Fig. 3 and Table S3). No significant difference was present between Mei1B and Mei2B in tillering number, grain width, and filled grain number per panicle (Fig. 3b and d). Compared to Mei1B, grain length, 1000 grain weight, and grain number per panicle of Mei2B were increased by 7.9%, 7.7%, and 25.5%, respectively. Although the seed setting rate of Mei2B was lower than Mei1B by 13.6%, its weight per plant was significantly increased by 14.9%. In view of the consistency of agronomic traits between the male sterile lines and their related maintainer lines (apart from, the only difference being in their fertility), this result only presented the histogram of the agronomic trait analysis of the maintainer lines concerned.

### Hybrids based on Mei2A carrying the edited gs3 allele had longer grains and yield than hybrids based on Mei1A with GS3 allele

**Fig. 4:**
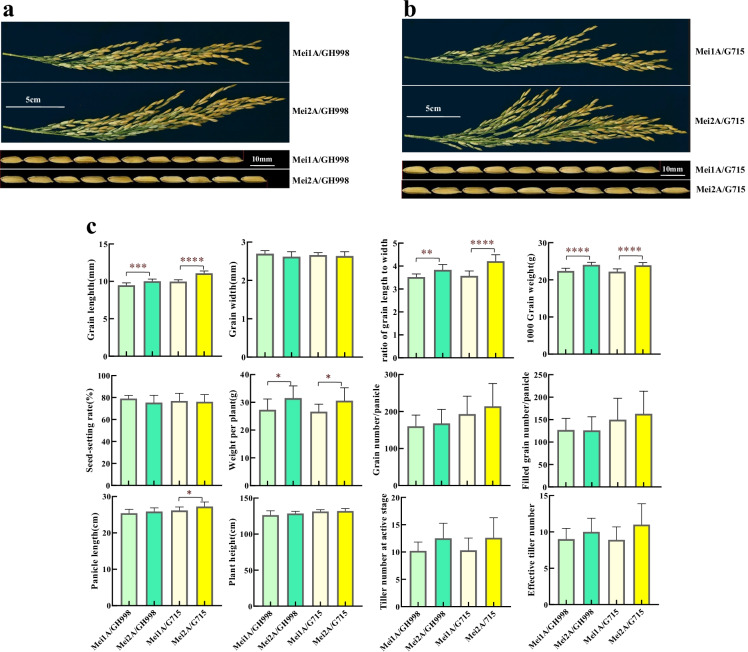
Comparisons of agronomic traits between the hybrids Mei2A/GH998 and Mei1A/GH998, Mei2A/G715 and Mei1A/G715. **a** Close-up views of panicle and grain length of combinations Mei1A/GH998 and Mei2A/GH998. **b** Close-up views of panicle and grain length of combinations Mei1A/GH998 and Mei2A/GH998. **c** Main agronomic trait comparisons between the hybrid combinations. *, **, **, and **** indicated the significant difference of studied traits at *p* levels 0.05, 0.01, 0.001, and 0.0001. Each *p* value for each trait was obtained from a *t* test between Mei1A/GH998 and Mei2A/GH998, Mei1A/G715, and Mei2A/G715 (*n* = 10 for each genotype)

Four hybrid combinations (Mei2A/GH998, Mei1A/GH998, Mei2A/G715, and Mei1A/G715) were developed by crossing the long-grain sterile line Mei2A and the original sterile line Mei1A with two restorer lines GH998 and G715, respectively. Compared with Mei1A/GH998, the hybrid combination Mei2A/GH998 showed 5.6%, 8.2%, 7.1%, and 15.4% increase in grain length, ratio of grain length to width, 1000 grain weight, and grain weight per plant, respectively. However, there were no significant differences between these two hybrid combinations in grain width, panicle length, plant height, grain number, filled grain number, seed-setting rate, and effective tillers (Table S3, Fig. 4). Similarly, there were no significant differences between the combinations of Mei2A/G715 and Mei1A/G715 in grain width, grain number, filled grain number, seed setting rate, plant height, and effective tiller. And Mei2A/G715 presented a significant increase in grain length, ratio of grain length to width, panicle length, 1000 grain weight, and weight per plant than Mei1A/G715 (Fig. 4). These traits were increased by 11.2%, 12.6%, 3.8% and 8.1%, 15.0%, respectively (Table S3, Fig. 4).

### Rice quality analysis

**Fig. 5:**
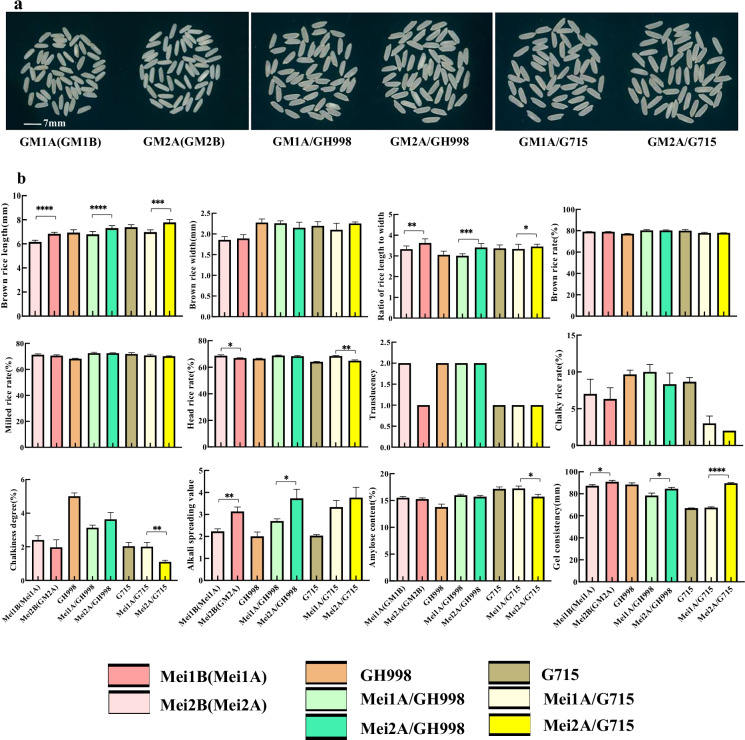
Rice grain quality comparisons between parents and their combinations. **a** Photographs of brown rice grains. **b** Comparison of grain quality related parameters. Mei1B and Mei1A were WT maintainer and male sterile line, separately; Mei2B and Mei2A were mutant maintainer and male sterile line, separately. GH998 and G715 were restorer lines. *, **, ***, and **** indicated significant difference of studied traits at *p* levels **0**.05, 0.01, 0.001, and 0.0001. Each *p* value for each trait was obtained from a *t* test between Mei1B(Mei1A) and Mei2B(Mei2A), Mei1A/GH998 and Mei2A/GH998, Mei1A/G715 and Mei2A/G715 (*n* = 10 for brown rice length, brown rice width, and ratio of rice length to width; *n* = 3 for the other genotypes except brown rice length, brown rice width, and ratio of rice length to width)

Since the grain length and ratio of grain length to width are highly related to the appearance quality of rice grain, its grain length, ratio of grain length to width, translucency grade, chalky rate, and chalkiness degree of the polished rice grain were then investigated in the hybrid parents and their hybrid combinations (Table S4, Fig. 5). Our results showed that Mei2B(Mei2A) and its combinations (Mei2A/GH998 and Mei2A/G715) had superior or a close appearance quality, compared to Mei1A/Mei1B and its combinations (Mei1A/GH998 and Mei1A/G715) (Fig. 5). In particular, the improved parental line Mei2B(Mei2A, *gs3*) had a 6.83 mm brown rice grain and the ratio of rice length to width of 3.61, which was close to the Simiao-type standard. We also analyzed the alkali spreading value, gel consistency, and amylose content traits for rice quality (Table S4). Compared with the original Mei1B(Mei1A), the alkali spreading value and gel consistency of the improved line Mei2B(Mei2A) were significantly increased. The two hybrids Mei1A/GH998 and Mei2A/GH998 showed significant difference in amylose content and alkali spreading value (Fig. 5). In the two G715 combinations, Mei2A/G715 exhibited lower amylose content and higher gel consistency, which indicated that the rice cooking quality of Mei2A/G715 was superior to Mei1A/G715. Although the head rice rate which reflects rice processing quality was decreased slightly in Mei2B(Mei2A) and Mei2A/G715 (Fig. 5), these results indicated that the overall appearance quality and rice qualities of Mei2B(Mei2A) and its combinations (Mei2A/GH998 and Mei2A/G715) had been improved, especially in Mei2A/G715. In addition, the genotypes of *GS3* and three quality-related genes (*Wx*^*b*^, *ALK*, *Chalk5*) were also consistent with rice quality testing; see Table 1 and Table S1.

**Table 1:** Genotypes of rice quality-related genes in hybrid parents and hybrid combinations

Variety	Fluorescence value and Genotype of *GS3*	Fluorescence value and Genotype of *Wx*	Fluorescence value and Genotype of *ALK*	Fluorescence value and Genotype of *Chalk5*
Mei1B(Mei1A)	*GS3*(FAM V)	*Wx*^*b*^(HEX V)	*ALK*(HEX V)	*Chalk5*(FAM V)
Mei2B(Mei2A)	*gs3*	*Wx*^*b*^(HEX V)	*ALK*(HEX V)	*Chalk5*(FAM V)
GH998	*gs3*(HEX V)	*Wx*^*b*^(HEX V)	*ALK*(HEX V)	*chalk5*(HEX V)
G715	*gs3* (HEX V)	*Wx*^*b*^(HEX V)	*ALK*(HEX V)	*Chalk5*(FAM V)
Mei1A/GH998	*GS3*/*gs3*	*Wx*^*b*^	*ALK*	*Chalk5*/*chalk5*
Mei1A/G715	*GS3*/*gs3*	*Wx*^*b*^	*ALK*	*Chalk5*
Mei2A/GH998	*gs3*	*Wx*^*b*^	*ALK*	*Chalk5*/ *chalk5*
Mei2A/G715	*gs3*	*Wx*^*b*^	*ALK*	*Chalk5*

## Discussion

The traits related to rice yield are important goals in the breeding of new varieties. Previous reports demonstrated that introducing the *gs3* gene into rice varieties substantially increased rice grain yield (Li et al. 2016; Shen et al. 2016; Chen et al. 2020). In this study, we found that Mei2B(*gs3*) had the significant increase in thousand grain weight (TGW) and grain yield per plant, consistent with the previous studies. Even though the seed setting rate of Mei2B(*gs3*) decreased, this was offset by the increases in grain number per panicle, filled grain per panicle, and TGW, making Mei2B to have a similar yield as Mei1B. The decreased seed setting rate of our improved maintainer line also occurred in previous studies (Shen et al. 2016; Chen et al. 2020). The source-sink-flow theory suggests that the increase in grain length, grain weight, and the total number of grains per panicle could have enhanced the “sink” capacity of the whole grain structure resulting in insufficient photosynthesis in individual plants and thus more empty grains or a lower seed setting rate (Shen et al. 2017). However, in contrast to the hybrids of our original parental line Mei1A(*GS3*), hybrids from of our improved line Mei2A(*gs3*) had a similar seed setting rate and an increased grain length, 1000 grain weight, and total yield per plant, thereby ensuring the overall stability of the yield (Table S3).The slender type Simiao or “silk seedling rice” is very popular in Southern China and has a higher market value. Through the introduction of a *gs3* allele, the grain length of the improved male sterile line Mei2A aligned with the grain length for Simiao, and this could greatly increase its market competitiveness. Indeed, not only the yield of their F_1_ was enhanced, but also the rice quality of their F_1_ reached the highly valued Simiao standard, when Mei2A was combined with a slender restorer G715. In addition, our data also indicated that the edited *gs3* allele improved the eating and cooking quality of the male sterile line and its hybrid combinations. Overall, our results demonstrated that the improvement of Mei1A through introducing a *gs3* allele via CRISPR/Cas9 technology indeed leads to a better grain appearance and yield in both the male sterile line itself and its hybrid combinations.

Rice quality traits of the combinations are usually determined or influenced by the male sterile line that has been used (Mao et al. 2006). It is important to breed a male sterile line which completely maintains the trait of male sterility. The CRISPR-Cas9-mediated genome editing technology can not only avoid some limitations in traditional crop breeding programs, but it can transfer the desired traits more quickly and accurately in this contemporary crop genetic improvement (Haque et al. 2018; Mishra et al. 2018). In our rice breeding project, the CRISPR/Cas9-mediated genome editing method was used to knock out the *GS3* gene in the maintainer line Mei1B, thereby successfully obtaining a *gs3* mutant that specifically resulted in a loss of function of the grain size gene. In the T_1_ mutant generation of Mei1B, a specific line with an increased grain length and homozygous mutated locus and no transgene was selected to generate the new maintainer line Mei2B. We obtained the new sterile lines Mei2A with the *gs3* allele through the improved maintainer line Mei2B and developed new hybrid combinations within 2 years in this study. In contrast, the conventional three-line rice breeding system requires lengthy cycles of backcrossings to evaluate and achieve stable sterile lines. Our study significantly shortened the breeding cycles to release new hybrid rice varieties.

We know that the restoration and conservation relationships of the heterosis within three-line hybrid rice breeding are far more complicated than that of the conventional rice production system, and such complications can restrict the genetic improvements of relevant traits (Zhou 1994; Gong et al. 2020). In our research, a genome editing tool CRISPR-Cas9 was initially adopted to knockout the GS3 gene of the maintainer line, and a molecular marker assisting selection skill was then used to further analyze the exact allelic effect of this target gene and the genotypes of quality-related genes. Detailed genetic information from both phenotyping and genotyping were applied together to guide our selection practices. Consequently, the combination of a molecular marker assisted selection (MAS) with genome editing technology proved to be significantly efficient regarding genetic improvement of the GS3 gene in the three-line hybrid rice production system. However, in this study, we specifically chose a strong restorer line GH998 to further evaluate the restoration effect on the improved male sterile line. Alongside this, another restorer line G715 was also selected and tested to make a long grain hybrid combination. Even though our test combination with the *gs3*/*gs3* allele overall produced a higher yield and quality (Fig. 4 and 5, Table 1). This indicates that the main QTL gene with negative regulation effects should gain more attention when selecting the genotype at the corresponding locus of the restorer lines. The maintainer line with a single gene knockout also had a strong positive restoration relationship with the restorer lines (Fig. 4), ensuring that potential genetic gain on high yield or high general combination ability (GCA) for individual cross combinations tested especially the restorer lines. In the future, joining the gene editing and molecular markers could reduce the challenges posed by phenotyping (Meuwissen et al. 2001; Poland and Rutkoski 2016) and obtain a more accurate genomic prediction in pre-emptive breeding programs (Emebiri et al. 2021).

## Supplementary Information

Below is the link to the electronic supplementary material.

Supplementary file1 (DOCX 82 KB)

## Data Availability

The data used in this study are available from the corresponding author on reasonable request.

## Abbreviations

CMS: Cytoplasmic male sterility
GH998: Guanghui 998
TGW: Thousand grain weight
LC: Low chalkiness
HC: High chalkiness
HASV: High alkali spreading value
WT: Wild type
G715: Gui715
FAM V: FAM value
HEX V: HEX value
LG: Long grain
LASV: Low alkali spreading value
SG: Short grain
AC: Amylose content
