# Supplementary material for: The genetic editing of GS3 via CRISPR/Cas9 accelerates the breeding of three-line hybrid rice with superior yield and grain quality
Source: Mol Breed. 2022 Apr 8;42(4):22. doi: 10.1007/s11032-022-01290-z (PMC10248666; doi:10.1007/s11032-022-01290-z)
Supplement: Supplementary file 1 — Supplementary file1 (DOCX 82 KB) [file 11032_2022_1290_MOESM1_ESM.docx]

Table S1. Primers used in this study

| Primer name | Sequence(5'-3') | Fluorescence value and corresponding genotype |
| --- | --- | --- |
| GS3-F1 | tccgccattcaaagcaaagc | - |
| GS3-R1 | gagtttaggtggagggacgc | - |
| GS3-F2 | acagtacttgctgtctagcttt | - |
| GS3-R2 | actcccaacgttcagaaattaaatg | - |
| GS3-Y1+ | cagtggtctcaggcaatgggcatgaaccaactcc | - |
| GS3-Y1- | cagtggtctcaaaacggagttggttcatgcccat | - |
| GS3-B1+ | cagtggtctcaggcatcaagactgtccagaaggc | - |
| GS3-B1- | cagtggtctcaaaacgccttctggacagtcttga | - |
| Yl-R+ | accggtaaggcgcgccgtagt | - |
| Pbw2- | gcgattaagttgggtaacgccaggg | - |
| Hyg-F | acgtctgtcgagaagtttctgatc | - |
| Hyg-R | agtcaatgaccgctgttatgc | - |
| Chalk5b-FT | gaaggtcggagtcaacggattagagagaagtgccaaggatctgt | HEX V:LC |
| Chalk5b-FC | gaaggtgaccaagttcatgctagagagaagtgccaaggatctgc | FAMV:HC |
| Chalk5b-R1 | tgcatctagctaccttcatttcg | - |
| RGs3-RT | gaaggtcggagtcaacggattcagcaggctggcttactctctt | FAM V: LG |
| RGs3-RG | gaaggtgaccaagttcatgctcagcaggctggcttactctctg | HEX V: SG |
| RGs3-F | acacatgcccatctccctcg | - |
| alk-Ftt | gaaggtgaccaagttcatgcttacaaggagagctggaggggtt | FAM V: LASV |
| alk-Fgc | gaaggtcggagtcaacggatttacaaggagagctggagggggc | HEX V:H ASV |
| alk-R | ctgaggtcctgcgacatgc | - |
| RWx-Fg | gaaggtgaccaagttcatgcttcatcaggaagaacatctgcaagg | FAM V: HAC |
| RWx-Ft | gaaggtcggagtcaacggatttcatcaggaagaacatctgcaagt | HEX V: L AC |
| RWx-R | ggaaaaacgagcaatgaaagatgc | - |

Table S2. Homozygous mutations of three transgenic plants and their grain length in T_0_ generation

| Accession  No. | Target sequences | Chromosome  No. | Sequence of mutation(5'-3') | Grain length(mm) |
| --- | --- | --- | --- | --- |
| P437-2 | cctcgaggaatccgatctcgcgg | 2 | CGCGAGATCGGATTCCTTCGAGGGTGAAATAAAT (insertion) | 10.33±0.36 |
| P437-6 | tgcagcatctggaggcagcgtgg | 2 | ATCCACGCTTGCCTCCAGATGCTGCAGAGAGGTTGACGAAT(insertion)  (insertion) | 10.22±0.45 |
| P437-13 | cctcgaggaatccgatctcgcgg | 2 | CGCGAGATCGGATTCCCTCGAGGGTGAAATAAAT (insertion) | 10.24±0.41 |
| Mei1B | - | - | - | 9.40±0.37 |

The inserted bases are highlighted in gray. All data are given as means ±s.e.m(n=10).

Table S3. Statistics of agronomic traits

| Variety | Grain length  (mm) | Gain width  (mm) | Ratio of grain length to width | Panicle length  (cm) | Grain number per panicle | Filled grain number per panicle | Seed-setting rate(%) | 1000 grain weight(g) | Effective tiller number | Tiller number at active stage | Plant height  (cm) | Weight per plant(g) |
| --- | --- | --- | --- | --- | --- | --- | --- | --- | --- | --- | --- | --- |
| Mei1B | 9.40±0.37 | 2.42±0.11 | 3.89±0.20 | 25.6±2.0 | 163.0±44.6 | 141.8±49.8 | 84.5±12.8 | 18.0±0.6 | 10.5±1.7 | - | 111.9±4.3 | 19.5±2.6 |
| Mei2B | 10.14±0.40  *** | 2.42±0.09 | 4.19±0.25** | 27.7±2.0* | 204.5±34.3* | 148.4±26.0 | 73.0±8.8* | 19.2±0.4* | 11.0±1.0 | - | 112.6±2.9 | 22.4±3.0* |
| GH998 | 9.59±0.36 | 2.65±0.13 | 3.63±0.25 | 22.9±1.5 | 161.6±11.7 | 129.8±12.2 | 80.5±8.6 | 21.7±0.5 | 8.6±0.9 | - | 104.0±2.8 | 26.9±3.0 |
| G715 | 11.30±0.37 | 2.62±0.14 | 4.32±0.33 | 27.0±1.3 | 227.8±52.7 | 181.8±37.7 | 80.2±4.3 | 22.5±0.7 | 7.4±1.1 | - | 123.2±1.6 | 28.0±6.2 |
| Mei1A/GH998 | 9.49±0.30 | 2.70±0.08 | 3.52±0.14 | 25.4±1.0 | 160.2±30.2 | 126.9±25.9 | 79.0±2.9 | 22.4±0.7 | 9.0±1.5 | 10.2±1.1 | 126.3±6.0 | 27.3±3.9 |
| Mei2A/GH998 | 10.02±0.29  *** | 2.62±0.13 | 3.83±0.23** | 25.9±1.0 | 167.8±38.2 | 126.0±30.4 | 75.4±6.6 | 24.0±0.8  **** | 10.0±2.0 | 12.5±2.8 | 128.6±3.1 | 31.5±4.4* |
| Mei1A/G715 | 9.96±0.26 | 2.66±0.06 | 3.74±0.13 | 26.2±1.1 | 193.0±48.3 | 149.8±47.7 | 76.8±7.1 | 22.2±1.0 | 8.9±1.6 | 10.3±1.7 | 131.3±2.5 | 26.6±2.7 |
| Mei2A/G715 | 11.08±0.31  **** | 2.64±0.11 | 4.21±0.28  **** | 27.2±1.2* | 214.0±61.6 | 163.1±50.2 | 76.1±6.6 | 24.0±0.6  **** | 11.0±2.8 | 12.6±3.7 | 132.1±2.9 | 30.6±4.7* |

All data are given as means ±s.e.m. *, **,***,**** indicate significant difference at *P* levels 0.05, 0.01, 0.001and 0.0001. Each p-value for each trait was obtained from a t-test between Mei1B and Mei2B, Mei1A/GH998 and Mei2A/GH998, Mei1A/G715 and Mei2A/G715(n=10 for each genotype of Mei1B and Mei2B, Mei1A/GH998 and Mei2A/GH998, Mei1A/G715 and Mei2A/G715; n=10 for grain length, gain width and ratio of grain length to width of GH998 and G715; n=5 for the other genotypes of GH998 and G715 except grain length, gain width and ratio of grain length to width).

Table S4. Analysis of rice quality for parents and their cross combinations

| Variety | Brown rice rate(%) | Milled rice rate(%) | Head rice rate(%) | Brown rice length  (mm) | Brown rice width (mm) | Ratio of rice length to width | Translucency | Chalky  rice rate(%) | Chalkiness degree(%) | Alkali spreading value | Gel consistency  (mm) | Amylose content (%) |
| --- | --- | --- | --- | --- | --- | --- | --- | --- | --- | --- | --- | --- |
| Mei1B(Mei1A) | 79.0±0.2 | 71.2±0.7 | 68.7±0.7 | 6.16±0.15 | 1.85±0.08 | 3.32±0.16 | 2±0 | 7.0±2.0 | 2.4±0.3 | 2.3±0.1 | 87.0±1.0 | 15.6±0.3 |
| Mei2B(Mei2A) | 78.9±0.3 | 70.5±0.7 | 66.9±0.3* | 6.83±0.12  **** | 1.89±0.09 | 3.61±0.20** | 1±0 | 6.3±1.5 | 2.0±0.6 | 3.1±0.2** | 91.0±1.3* | 15.3±0.2 |
| GH998 | 76.9±0.4 | 68.2±0.4 | 66.5±0.3 | 6.93±0.24 | 2.27±0.09 | 3.05±0.18 | 2±0 | 9.7±0.6 | 5.0±0.2 | 2.0±0.2 | 88.0±1.5 | 13.9±0.6 |
| G715 | 80.0±1.1 | 71.8±1.0 | 64.0±0.4 | 7.38±0.21 | 2.19±0.10 | 3.37±0.16 | 1±0 | 8.7±0.6 | 2.0±0.2 | 2.0±0.1 | 66.8±0.3 | 17.2±0.4 |
| Mei1A/GH998 | 80.3±0.9 | 72.4±0.8 | 68.8±0.4 | 6.63±0.25 | 2.26±0.06 | 2.94±0.10 | 2±0 | 10.0±1.0 | 3.1±0.2 | 2.7±0.1 | 78.3±0.3 | 16.0±0.2 |
| Mei2A/GH998 | 80.2±0.6 | 72.4±0.4 | 68.3±0.6 | 7.10±0.21  **** | 2.21±0.13 | 3.21±0.19*** | 2±0 | 8.3±1.5 | 3.6±0.4 | 3.7±0.4* | 84.5±1.3* | 15.7±0.3 |
| Mei1A/G715 | 77.9±0.6 | 70.7±1.0 | 68.4±0.6 | 6.97±0.19 | 2.10±0.16 | 3.34±0.22 | 1±0 | 3.0±1.0 | 2.0±0.3 | 3.3±0.3 | 67.3±0.8 | 17.2±0.5 |
| Mei2A/G715 | 77.9±0.3 | 70.1±0.4 | 64.9±0.7  ** | 7.31±0.17  *** | 2.08±0.06 | 3.52±0.11* | 1±0 | 2.0±0 | 1.1±0.1** | 3.8±0.5 | 89.5±0.5**** | 15.7±0.5* |

All data are given as means ±s.e.m. *, **,***,**** indicate Significant difference at *P* levels 0.05, 0.01, 0.001and 0.0001. Each p-value for each trait was obtained from a t-test between Mei1B and Mei2B, Mei1A/GH998 and Mei2A/GH998, Mei1A/G715 and Mei2A/G715(n=10 for brown rice length, brown rice width and ratio of rice length to width; n=3 for the other genotypes except brown rice length, brown rice width and ratio of rice length to width ).


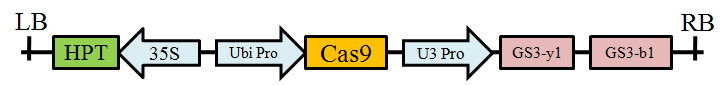


Fig. S1 Schematic map of the recombinant plasmid CRISPR-Cas9 -GS3. The inserted fragment region includes knockout targets GS3-y1 and GS3-b1 activated by U3 promoter, *Cas9* gene activated by ubiquitin promoter and hygromycin phosphotransferase gene activated by 35S promoter. LB, T-DNA left border sequence; RB, T-DNA right border sequence.


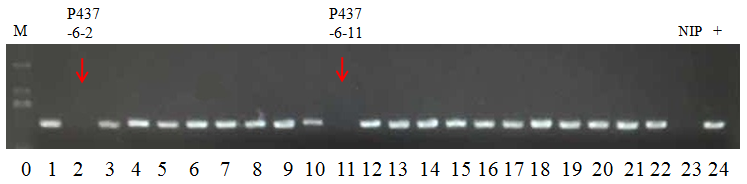


Fig. S2 PCR identification of the transgene-free transgenic plants. A primer pair Hyg-F/ Hyg-R was used to amplify a fragment of HPT (hygromin phosphotransferase) gene. Lane 0: M, Marker 2000. Lane 1-22, individual seedlings of P437-6 mutant; Lane23, NIP, Negative control(Nipponbare); Lane, 24, +, positive control of transgenic line; Lanes with amplified PCR fragment indicated transgene positive. Lanes without amplified PCR fragment indicated transgene-free. Two transgene-free mutants P437-6-2 and P437-6-11 were pointed with a red arrow.
